# Between-cow variation in milk fatty acids associated with methane production

**DOI:** 10.1371/journal.pone.0235357

**Published:** 2020-08-06

**Authors:** J. de Souza, H. Leskinen, A. L. Lock, K. J. Shingfield, P. Huhtanen

**Affiliations:** 1 Department of Animal Science, Michigan State University, East Lansing, Michigan, United States of America; 2 Milk Production, Production Systems, Natural Resources Institute Finland (Luke), Jokioinen, Finland; 3 Department of Agricultural Research for Northern Sweden, Swedish University of Agricultural Sciences, Umeå, Sweden; The University of Sydney, AUSTRALIA

## Abstract

We evaluated the between-cow (b-cow) variation and repeatability in omasal and milk fatty acids (FA) related to methane (CH_4_) emission. The dataset was originated from 9 studies with rumen-cannulated dairy cows conducted using either a switch-back or a Latin square design. Production of CH_4_ per mole of VFA (Y_CH_4_VFA) was calculated based on VFA stoichiometry. Experiment, diet within experiment, period within experiment, and cow within experiment were considered as random factors. Empirical models were developed between the variables of interest by univariate and bivariate mixed model regression analysis. The variation associated with diet was higher than the b-cow variation with low repeatability (< 0.25) for milk odd- and branch-chain FA (OBCFA). Similarly, for de novo synthesized milk FA, diet variation was ~ 3-fold greater than the b-cow variation; repeatability for these FA was moderate to high (0.34–0.58). Also, for both *cis*-9 C18:1 and *cis*-9 *cis*-12 *cis*-15 C18:3 diet variation was more than double the b-cow variation, but repeatability was moderate. Among the de novo milk FA, C4:0 was positively related with stoichiometric Y_CH_4_VFA, while for OBCFA, *anteiso* C15:0 and C15:0 were negatively related with it. Notably, when analyzing the relationship between omasal FA and milk FA we observed positive intercept estimates for all the OBCFA, which may indicate endogenous post-ruminal synthesis of these FA, most likely in the mammary gland. For milk *iso* C13:0, *iso* C15:0, *anteiso* C15:0, and C15:0 were positively influenced by omasal proportion of their respective FA and by energy balance. In contrast, the concentration of milk C17:0, *iso* C18:0, C18:0, *cis*-11 C18:1, and *cis*-9 *cis*-12 *cis*-15 C18:3 were positively influenced by omasal proportion of their respective FA but negatively related to calculated energy balance. Our findings demonstrate that for most milk FA examined, a larger variation is attributed to diet than b-cow differences with low to moderate repeatability. While some milk FA were positively or negatively related with Y_CH_4_VFA, there was a pronounced effect of calculated energy balance on these estimates. Additionally, even though OBCFA have been indicated as markers of rumen function, our results suggest that endogenous synthesis of these FA may occur, which therefore, may limit the utilization of milk FA as a proxy for CH_4_ predictions for cows fed the same diet.

## Introduction

Enteric methane (CH_4_) production is one of the main sources of green-house gas (GHG) emissions from dairy production systems, and enteric CH_4_ production is among the main targets of GHG mitigation practices for the dairy industry [1]. Therefore, mitigating enteric CH_4_ emissions is an approach for improving sustainability and profitability of dairy production systems [2]. Direct measurements of CH_4_ are difficult to perform under regular farm conditions; therefore, the development of prediction equations to estimate CH_4_ output has gained significance [3–5]. Changes in absorbed fatty acid (FA) composition affected by ruminal metabolism and microbial synthesis of FA can affect milk FA composition [6], and may therefore predict changes in the ruminal fermentation associated with CH_4_ emissions. It is well established that de novo synthesis in the mammary gland yields short and medium-chain FA (4 to 14 carbons) and a portion of the 16-carbon FA derived from acetate and to a lesser extent BHBA. The remaining 16-carbon and all of the longer-chain FA (greater than 16 carbons) are taken up from the circulating plasma pool originated from absorption from the digestive tract or mobilization from body reserves. Additionally, odd- and branched-chain FA (**OBCFA**) in milk fat are largely derived from bacteria leaving the rumen [7] and have been suggested as potential biomarkers for rumen function [6]. The potential utilization of milk FA to predict CH_4_ has been studied from direct in vivo measurements [8, 9] and from meta-analysis approaches [10, 11]. These models have selected several different FA as potential CH_4_ predictors, which indicates an important influence of other dietary and animal factors influencing these estimates.

Variation in CH_4_ production has been also attributed to animal factors [3, 4]. Studies conducted in sheep have shown that the variation in ruminal digesta retention time or passage rate is related to CH_4_ emissions, with high CH_4_ emitters having a larger rumen volume and digesta pools than low CH_4_ emitters [12]. Recently, Cabezas-Garcia et al. [13] reported that variables related to animal physiology, such as variation in digesta retention time, can explain most of the between-animal variations in CH_4_ production. Only small variations were observed in rumen fermentation variables, especially stoichiometric Y_CH_4_VFA, suggesting a minor contribution of the rumen microbiome to CH_4_ production. Since some studies have indicated that potentially several individual milk FA can be used to predict CH_4_ emission in lactating dairy cows, the examination of between-animal differences in a data set originating from variations in digestion physiology and different diets is important. Also, because animal variation is likely to be under genetic control, one option to mitigate CH_4_ emissions that has been suggested is to select for animals that emit less. Heritability of some major milk FA have been previously determined [14, 15]. However, although a large range in the heritability of specific milk FA were reported [14, 15], they did not report heritability and variation in milk FA directly related with rumen function (i.e. OBCFA and *trans*-FA). Since potentially several individual milk FA can be used to predict CH_4_ emission in lactating dairy cows, the examination of between-animal differences in a data set originating from variations in digestion physiology and different diets is important. Additionally, integration of data related to rumen function with nutrient outflow and milk output may allow for a better understanding of the variables involved in the observed between-animal and between-diets variation. The objective of our meta-analysis was to evaluate b-cow variation and repeatability in omasal fatty acids and milk fatty acids associated with CH_4_ emission.

## Materials and methods

### Data

The dataset was originated from 9 studies [16–25], 29 cows and 33 different diets of rumen cannulated Nordic red dairy cows, conducted using either a Latin square or switch-back design conducted in the Finland (S1 Table). These studies evaluated a wide range of different dietary condition including different forages strategies, forage conservation method, forage: concentrate levels, and supplementation with different fatty acids. The mean forage-to-concentrate ratio of the diets was 57:43 on a DM basis. The concentrate supplements consisted principally of cereal grains, fibrous by-products from the food industry, and protein supplements, typically canola meal. In some studies diets were supplemented with sunflower oil, rapeseed oil, linseed oil or fish oil. Formic acid-treated grass silage was the main forage source, but red clover silage, extensively fermented grass silage (no additives), fresh chopped grass, and barn-dried hay were used in some studies. The diets were fed ad libitum or at 90 to 95% of ad libitum intake as TMR or fixed amounts of concentrate with forage ad libitum. The complete data set consisted of 135 cow/period observations, which were the experimental unit. A minimum pre-condition for inclusion of a study in the meta-analysis was that feed intake, BW, milk production data, fermentation parameters, omasal FA, and milk FA profile were available.

Individual cow intakes and milk yield were recorded daily throughout the experiment, but only measurements for the last 4–7 d were used for analysis. Samples of milk were collected from each cow over 4 consecutive milkings. Milk samples treated with preservative (bronopol; Valio Ltd., Helsinki, Finland) and were stored at 4°C until analyzed for milk components (MilkoScan 133B analyzer; Foss Electric A/S, Hillerød, Denmark). Unpreserved milk samples were also collected at the same time, stored immediately at −20°C, and composited according to milk yield until analyzed for FA composition. Body weight was measured weekly.

Diet digestibility was determined by total feces collection over 4 to 5 days. Digesta flow measurements were conducted using the omasal sampling technique [26] with a triple-marker system [27] based on Co-EDTA or Cr-EDTA, Yb-acetate, and Cr-mordanted straw or indigestible NDF as markers for liquid, small, and large particles, respectively. Rumen fluid samples (n = 7 or 8) were collected at 1.5 intervals (approximately 500 mL) starting just before morning feeding at 0600 h through rumen cannula using a vacuum pump and flexible tube and analyzed for pH, VFA and ammonia N concentrations [28]. Spot samples (500 mL) of digesta entering the omasal canal were collected 3 times daily at 4-h intervals during 4 consecutive days, to cover a 12-h period that was considered representative of the entire feeding cycle composited, and separated into large particle, small particle, and liquid phases. Each phase was freeze-dried and stored at −20°C, whereas subsamples of each fraction collected for FA analysis were stored at −20°C. Metabolizable energy content of experimental diets was calculated from the concentration of digestible nutrients [0.016 × digestible OM in DM (g/kg); Ministry of Agriculture, Fisheries and Food, 1975] determined by total fecal collection. The energy requirement (MJ/d) for maintenance and milk production was calculated as [BW (kg ^0.75^) × 0.515 + ECM yield (kg/d) × 5.15]; [29]. Energy balance was calculated as ME intake–ME maintenance–ME production, all expressed as MJ/d.

The VFA ratios acetate/propionate and propionate/butyrate were calculated, and the lipogenic:glucogenic ratio of VFA was determined as (acetate + butyrate)/ propionate. Production of CH_4_ per mole of VFA (Y_CH_4_VFA) was calculated based on VFA stoichiometry [30] as:

$$\text{Y}\_{\text{CH}}_{4}\text{VFA}(\text{mmol}/\text{mol}\;\text{of}\;\text{VFA})=0.5\times {\text{C}}_{2}–0.25\times {\text{C}}_{3}+0.5\times {\text{C}}_{4},$$

where C_2_, C_3_, and C_4_ are molar proportions (mmol/mol) of acetate, propionate, and butyrate, respectively, in the sum of these VFA.

Total lipid in milk, oil supplements, freeze-dried feed samples and omasal digesta were converted to FA methyl esters (**FAME**) using standard methods [31, 32]. The FAME were quantified using a gas chromatograph equipped with a flame-ionization detector and a CP-Sil 88 column (100 m x 0.25 mm id., 0.2 μm film thickness; Agilent Technologies, Santa Clara, CA).

### Statistical analysis

All analysis were performed using the MIXED procedure of SAS (SAS version 9.3, SAS Institute Inc., Cary, NC). Variance components of the selected variables was calculated considering random factors of experiment (Exp), diet within experiment [Diet (Exp)], period within experiment [Period (Exp)], and cow within experiment [Cow (Exp)]. Covariance structure was defined in the model using the TYPE = VC (variance components) option in the RANDOM statement. The standard deviation and coefficient of variation for each factor were calculated as the square root of the variance estimate and standard deviation divided by the respective mean value of each factor.

Repeatability values (Rep) for the most relevant variables associated with enteric CH_4_ production were calculated as

$$\text{Rep}=\sigma_{\text{Cow}}^{2}/(\sigma_{\text{Cow}}^{2}+\sigma_{\text{Residual}}^{2}),$$

where $\sigma_{\text{Cow}}^{2}$ and $\sigma_{\text{Residual}}^{2}$ are Cow (Exp) and residual variances, respectively. Repeatability values provide an estimate of the correlation between values from consecutive samples on the same cow, on the same diet, and within the same period of the same experiment. For this study, repeatability was classified as low (<0.25), moderate (0.26–0.50) and high (>0.50).

Empirical models were developed between the variables of interest regarding their biological value by regression analysis within the MIXED procedure of SAS, using the following model:

$$Y_{ij}=B_{0}+B_{1}X_{1ij}+b_{0}+b_{1}X_{1ij}+e_{ij},$$

where Y_ij_ = the expected value for the dependent variable Y observed at level of j of the independent variable X in study i; B_0_ = the overall intercept (fixed effect); b_0_ = the random effect of study i on the intercept (i = 1, …, 9); B_1_ = the regression coefficient of Y on X_1_ of Y across all studies (fixed effects), X_1ij_ = value j of the continuous variable X_1_ in study i; bi = the random effect of study i on the regression coefficient of Y on X1 in study i (i = 1, …, 9), and eij = the residual error.

The models included 2 random statements: a random intercept and slope of X_1_ with SUBJECT = Diet (Exp), and a random intercept with SUBJECT = Period (Exp), using the TYPE = VC as the covariance structure for both random statements. The method = ML (maximum likelihood) statement was used in the PROC MIXED model syntax. Only one random independent variable was used to avoid overparameterized models and improve convergence [33].

## Results

### Data description

Mean and ranges of nutrient intake, production responses and rumen fermentation parameters are presented in Table 1. Despite the large differences in diet composition, rumen pH and the proportions of major rumen VFA did not vary greatly compared with the proportions of minor VFA. The large variation in intake of total FA is related to several studies in this data set supplemented different sources of FA.

**Table 1:** Minimum, maximum, mean, and standard deviation values for milk yield, milk composition, energy balance, nutrient intake, rumen fermentation, digestibility, and predicted CH_4_ in the data set.

Variable	N	Mean	SD	Minimum	Maximum
Milk yield, kg/d	135	26.5	6.85	5.00	41.8
ECM, kg/d	135	25.8	6.46	5.20	40.6
Milk fat content, %	135	3.83	0.623	1.65	5.18
Milk protein content, %	135	3.37	0.497	2.13	5.80
BW, kg	135	616	48.7	490	770
DIM	38	132	63.9	38	304
ME intake, MJ/d	135	211	34.1	147	306
ME balance, MJ/d	135	2.85	26.282	-62.3	71.2
Intake, kg/d					
Forage DM	135	10.6	2.36	2.46	17.2
Concentrate DM	135	8.2	2.58	2.64	17.3
Total DM	135	18.9	2.97	12.1	25.8
OM	135	17.4	2.78	11.0	24.1
CP	135	2.9	0.56	1.75	4.55
NDF	135	7.5	1.49	4.02	12.2
iNDF	135	1.32	0.260	0.68	1.91
FA	135	0.71	0.331	0.27	1.66
Digestibility coefficient					
OM	135	0.74	0.031	0.67	0.82
NDF	135	0.65	0.072	0.41	0.78
CH_4_VFA	134	352	18.2	275	382
Total VFA, mmol/L	134	109	12.7	78.4	149
Molar proportion, mmol/mol					
Acetate (A)	134	648	26.2	569	695
Propionate (P)	134	189	22.9	150	287
Butyrate (B)	134	124	15.9	79.8	195
Isobutyrate	134	8.0	1.52	4.44	12.6
Valerate	134	13.6	3.22	4.14	21.1
Isovalerate	134	12.4	3.81	5.44	27.9
VFA ratio					
A:P	134	3.48	0.481	2.04	4.57
(A+B)/P	134	4.15	0.572	2.32	5.37
Rumen pH	134	6.47	0.302	5.73	7.22

Mean and ranges of proportion of omasal FA are presented in Table 2. As expected, the predominant FA in the omasum was C18:0 with a wide range of it as a proportion of omasal FA. Also, C16:0, *trans*-11 C18:1, *cis*-9 C18:1, and *cis*-9, *cis*-12 C18:2 represented the main FA present in the omasum. Variation in the proportion of OBCFA was a similar across all of these FA.

**Table 2:** Minimum, maximum, mean, and standard deviation values for selected fatty acids (FA) content in omasal digesta in the data set.

Variable	N	Mean	SD	Minimum	Maximum
Selected FA, g 100g/FA					
C13:0 iso	134	0.04	0.029	0.01	0.15
C13:0 anteiso	104	0.02	0.019	0.01	0.09
C15:0 iso	135	0.28	0.190	0.05	0.87
C15:0 anteiso	123	0.51	0.322	0.13	1.29
C15:0	123	0.79	0.339	0.31	1.57
C16:0 iso	135	0.20	0.143	0.02	0.68
C16:0	131	11.6	2.71	6.53	15.9
C17:0 iso	135	0.23	0.106	0.05	0.49
C17:0 anteiso	126	0.20	0.107	0.04	0.50
C17:0	135	0.46	0.160	0.17	0.90
C18:0 iso	135	0.06	0.023	0.01	0.15
C18:0	135	50.0	14.01	7.23	72.6
C18:1, trans-10	135	1.91	3.825	0.31	23.2
C18:1, trans-11	135	5.17	3.326	1.40	19.2
C18:1, cis-9	135	3.07	1.245	0.79	8.53
C18:1, cis-11	135	0.71	0.298	0.29	1.81
C18:2, cis-9 cis-12	135	2.29	1.370	0.21	5.00
C18:2, cis-9 trans-11	135	0.66	0.560	0.08	2.83
C18:3, cis-9 cis-12 cis-15	135	1.06	0.768	0.12	3.44

Mean and ranges of milk FA profiles are presented in Table 3. As expected the major FA in milk fat were C16:0, *cis*-9 C18:1, and C18:0. Although preformed FA were the major FA in milk fat, large variation in the summation of de novo FA, mixed FA and preformed milk FA was also observed. There was a similar variation among the milk OBCFA with *iso* C17:0 showing the greatest variation. Also, the large variation in milk *trans*-10 C18:1 and *trans*-11 C18:1 is related to several studies in this data set which had diets that induced milk fat depression.

**Table 3:** Minimum, maximum, mean, and standard deviation values for selected milk fatty acids (FA) in the data set.

Variable	N	Mean	SD	Minimum	Maximum
Selected FA, g 100g/FA					
C4:0	135	3.28	0.677	1.07	4.57
C6:0	135	1.88	0.414	0.62	2.66
C8:0	135	1.10	0.291	0.31	1.64
C13:0 iso	135	0.03	0.006	0.01	0.05
C13:0 anteiso	131	0.01	0.004	0.01	0.03
C15:0 iso	135	0.22	0.049	0.09	0.35
C15:0 anteiso	135	0.43	0.099	0.23	0.63
C15:0	135	0.99	0.267	0.48	1.78
C16:0 iso	132	0.23	0.051	0.16	0.42
C16:0	135	27.2	6.112	17.7	40.3
C17:0 iso	135	0.16	0.075	0.06	0.51
C17:0 anteiso	135	0.27	0.096	0.15	0.71
C17:0	135	0.51	0.110	0.27	0.80
C18:0 iso	135	0.05	0.015	0.02	0.11
C18:0	135	10.8	3.832	2.43	19.2
C18:1, trans-10	135	1.01	2.178	0.08	12.7
C18:1, trans-11	135	1.91	1.696	0.51	8.67
C18:1, cis-9	135	18.6	6.39	7.18	34.4
C18:1, cis-11	135	0.60	0.203	0.31	1.43
C18:2, cis-9 trans-11	135	0.78	0.663	0.19	3.90
C18:3, cis-9 cis-12 cis-15	135	0.50	0.182	0.22	1.29
<16-carbon	132	25.9	5.03	14.2	34.9
16-carbon	132	29.9	6.15	19.6	43.5
>16-carbon	132	44.2	10.46	24.8	64.2

^1^ De novo FA originate from mammary de novo synthesis (<16 carbons), preformed FA originated from extraction from plasma (>16 carbons), and mixed FA originate from both sources.

### Variance components

In general, the effect of experiment (Exp) was the largest source of variation observed in the data set (not shown). The variance components for rumen fermentation patterns are shown in Table 4. For the molar proportions of acetate and propionate, we observed that the variation related to diet was more than double the b-cow variation, with moderate repeatability, whereas for butyrate and isobutyrate the diet and b-cow variance components were similar. Diet and b-cow variance components were similar for both rumen pH and total VFA, and they were more repeatable than molar proportions of individual VFA. The b-cow variation for Y_CH_4_VFA was only 0.010, and repeatability was low.

**Table 4:** Variance component estimates for methane estimate and rumen fermentation parameters in dairy cows.

Variable		Estimate	SE	Z value^1^	SD^2^	CV^3^	Rep^4^
CH_4_VFA, mmol/mol	Diet (Exp)	110	38.9	<0.01	10.5	0.03	0.22
	Cow (Exp)	23.6	14.54	0.05	4.86	0.01	
	Residual	84.7	14.96	<0.01	9.20	0.03	
TVFA, mmol	Diet (Exp)	24.0	9.91	<0.01	4.90	0.05	0.46
	Cow (Exp)	30.9	11.03	<0.01	5.56	0.05	
	Residual	36.9	7.35	<0.01	6.07	0.06	
Acetate, mmol/mol	Diet (Exp)	416	130.0	<0.01	20.4	0.03	0.37
	Cow (Exp)	56.6	23.27	<0.01	7.52	0.01	
	Residual	97.8	19.43	<0.01	9.89	0.02	
Propionate, mmol/mol	Diet (Exp)	151	46.2	<0.01	12.3	0.07	0.28
	Cow (Exp)	40.7	20.44	0.02	6.38	0.03	
	Residual	103	23.1	<0.01	10.2	0.05	
Butyrate, mmol/mol	Diet (Exp)	30.9	19.33	0.05	5.56	0.05	0.10
	Cow (Exp)	15.6	16.20	0.17	3.95	0.03	
	Residual	147	23.2	<0.01	12.1	0.10	
Isobutyrate, mmol/mol	Diet (Exp)	0.19	0.125	0.07	0.43	0.05	0.02
	Cow (Exp)	0.02	0.085	0.42	0.13	0.02	
	Residual	0.91	0.184	<0.01	0.96	0.12	
Isovalerate, mmol/mol	Diet (Exp)	2.73	1.002	<0.01	1.65	0.13	0.35
	Cow (Exp)	1.46	0.586	<0.01	1.21	0.10	
	Residual	2.76	0.541	<0.01	1.66	0.13	
Ratio^5^	Diet (Exp)	0.10	0.039	<0.01	0.32	0.08	0.08
	Cow (Exp)	0.01	0.014	0.23	0.10	0.03	
	Residual	0.12	0.020	<0.01	0.34	0.09	
BCVFA^6^	Diet (Exp)	3.09	1.276	<0.01	1.76	0.09	0.23
	Cow (Exp)	1.51	0.792	0.03	1.23	0.06	
	Residual	5.16	1.024	<0.01	2.27	0.11	
pH	Diet (Exp)	0.02	0.007	<0.01	0.14	0.02	0.55
	Cow (Exp)	0.02	0.006	<0.01	0.14	0.02	
	Residual	0.02	0.003	<0.01	0.12	0.02	

^1^Probability of Z-value.

^2^Calculated as the square root of the variance component estimate.

^3^Calculated as SD divided by the respective mean value of the variable.

^4^Rep = σ^2^ Cow/(σ^2^ Cow + σ^2^ Residual).

^5^ Ratio = (Acetate +Butyrate) / (Propionate + Valerate).

^6^ BCVFA = Isovalerate + Isobutyrate.

The variance components for the proportion of omasal FA are presented in Table 5. For the OBCFA including *iso* C13:0, *anteiso* C13:0, *iso* C15:0, *iso* C16:0, *iso* C17:0, *anteiso* C17:0, C17:0 and *iso* C18:0 the variation associated with diet was greater than the between-cow variation with low repeatability. For *anteiso* C15:0 and C15:0 the variation associated with diet was also greater than the between-cow variation with moderate repeatability. Although the variation associated with diet for C16:0 was more than double the between-cow variation, this FA had the highest repeatability. C18:0, *cis*-9 C18:1 and *cis*-11 C18:1 had low repeatability, and the variation associated with diet was greater than the between-cow variation. Similarly, for *trans*-10 C18:1, *trans*-11 C18:1, *cis*-9, *cis*-12 C18:2 and *cis*-9 *cis*-12 *cis*-15 C18:3 diet variation was greater than between-cow variation with low repeatability, whereas *cis*-9 *trans*-11 C18:2 had diet and between-cow variance components similar and high repeatability.

**Table 5:** Variance component estimates of omasal fatty acids (FA) in dairy cows.

Variable		Estimate	SE	Z value^1^	SD^2^	CV^3^	Rep^4^
Selected FA, g 100g/FA							
C13:0 iso	Diet (Exp)	0.0001	0.00004	<0.01	0.01	0.30	0.04
	Cow (Exp)	0.00002	0.000007	0.37	0.002	0.04	
	Residual	0.0001	0.00001	<0.01	0.008	0.21	
C13:0 anteiso	Diet (Exp)	0.0016	0.00081	0.02	0.04	1.68	0.17
	Cow (Exp)	0.0006	0.00052	0.11	0.02	1.02	
	Residual	0.003	0.0006	<0.01	0.05	2.24	
C15:0 iso	Diet (Exp)	0.006	0.0020	<0.01	0.08	0.28	0.05
	Cow (Exp)	0.0002	0.00031	0.30	0.01	0.05	
	Residual	0.003	0.0005	<0.01	0.05	0.19	
C15:0 anteiso	Diet (Exp)	0.39	0.151	<0.01	0.63	1.23	0.30
	Cow (Exp)	0.18	0.092	0.02	0.42	0.83	
	Residual	0.41	0.082	<0.01	0.64	1.25	
C15:0	Diet (Exp)	0.72	0.241	<0.01	0.85	1.08	0.31
	Cow (Exp)	0.17	0.083	0.02	0.42	0.53	
	Residual	0.40	0.084	<0.01	0.63	0.80	
C16:0 iso	Diet (Exp)	0.003	0.0011	<0.01	0.06	0.29	0.02
	Cow (Exp)	0.0001	0.00034	0.41	0.009	0.04	
	Residual	0.003	0.0006	<0.01	0.06	0.29	
C16:0	Diet (Exp)	11.9	3.60	<0.01	3.45	0.30	0.53
	Cow (Exp)	2.46	0.841	<0.01	1.57	0.14	
	Residual	2.14	0.433	<0.01	1.46	0.13	
C17:0 iso	Diet (Exp)	0.003	0.0010	<0.01	0.06	0.24	0.02
	Cow (Exp)	0.00002	0.000108	0.44	0.004	0.02	
	Residual	0.001	0.0002	<0.01	0.03	0.14	
C17:0 anteiso	Diet (Exp)	0.003	0.0009	<0.01	0.05	0.27	0.10
	Cow (Exp)	0.0002	0.00021	0.21	0.01	0.07	
	Residual	0.002	0.0003	<0.01	0.04	0.20	
C17:0	Diet (Exp)	0.01	0.003	<0.01	0.10	0.21	0.21
	Cow (Exp)	0.0002	0.00014	0.05	0.02	0.03	
	Residual	0.0009	0.00015	<0.01	0.03	0.06	
C18:0 iso	Diet (Exp)	0.0002	0.00007	<0.01	0.01	0.26	0.05
	Cow (Exp)	0.00005	0.000010	0.32	0.002	0.04	
	Residual	0.0001	0.00002	<0.01	0.009	0.16	
C18:0	Diet (Exp)	83.5	25.11	<0.01	9.14	0.18	0.06
	Cow (Exp)	0.95	1.452	0.26	0.98	0.02	
	Residual	15.4	2.50	<0.01	3.93	0.08	
C18:1, trans-10	Diet (Exp)	10.6	3.15	<0.01	3.26	1.71	0.15
	Cow (Exp)	0.29	0.201	0.08	0.54	0.28	
	Residual	1.67	0.321	<0.01	1.29	0.68	
C18:1, trans-11	Diet (Exp)	5.68	1.752	<0.01	2.38	0.46	0.13
	Cow (Exp)	0.24	0.193	0.10	0.49	0.10	
	Residual	1.64	0.272	<0.01	1.28	0.25	
C18:1, cis-9	Diet (Exp)	0.91	0.283	<0.01	0.95	0.31	0.14
	Cow (Exp)	0.04	0.031	0.10	0.21	0.07	
	Residual	0.26	0.052	<0.01	0.51	0.17	
C18:1, cis-11	Diet (Exp)	0.05	0.023	<0.01	0.23	0.32	0.09
	Cow (Exp)	0.001	0.0021	0.20	0.04	0.05	
	Residual	0.02	0.003	<0.01	0.12	0.18	
C18:2, ci9- cis-12	Diet (Exp)	0.48	0.153	<0.01	0.69	0.30	0.12
	Cow (Exp)	0.02	0.022	0.13	0.14	0.06	
	Residual	0.14	0.031	<0.01	0.38	0.16	
C18:2, cis-9 trans-11	Diet (Exp)	0.02	0.007	<0.01	0.13	0.20	0.53
	Cow (Exp)	0.02	0.008	<0.01	0.16	0.24	
	Residual	0.02	0.004	<0.01	0.15	0.22	
C18:3, cis-9 cis-12 cis-15	Diet (Exp)	0.14	0.041	<0.01	0.37	0.35	0.20
	Cow (Exp)	0.005	0.0030	0.07	0.07	0.06	
	Residual	0.02	0.004	<0.01	0.14	0.13	

^1^Probability of Z-value.

^2^Calculated as the square root of the variance component estimate.

^3^Calculated as SD divided by the respective mean value of the variable.

^4^Rep = σ^2^ Cow/(σ^2^ Cow + σ^2^ Residual).

The variance components for milk FA are presented in Table 6. Milk FA can be classified in three groups because they are derived from 2 sources: de novo synthesis in the mammary gland (< 16 carbon FA) and originating from extraction from plasma (> 16 carbon FA). Mixed source FA (16-carbon milk FA) can be originate from both pools. Interestingly, for the summation of milk FA by source (de novo, mixed and preformed), the diet variation was greater than the b-cow variation, but these groups of FA had moderate to high repeatability. For de novo milk FA C4:0, C6:0 and C8:0 the diet variation was approximately 3-fold greater than the b-cow variation; repeatability for these FA was moderate to high. For OBCFA including *iso* C13:0, *anteiso* C13:0, *iso* C16:0, *iso* C17:0, the diet variation was 2 to 3-fold the b-cow variation with low repeatability. Although diet variation was greater than b-cow variation for *iso* C15:0, C15:0, *anteiso* C15:0, *anteiso* C17:0, C17:0, and *iso* C18:0, these FA had moderate repeatability. Similarly, diet variation for C16:0 was more than double the b-cow variation, though this FA had the highest repeatability. C18:0, and *cis*-11 C18:1 had moderate repeatability, and the variation related to diet was greater than the b-cow variation. For both preformed milk FA (*cis*-9 C18:1 and *cis*-9, *cis*-12, *cis*-15 C18:3) diet variation was greater than the b-cow variation, but repeatability was moderate. For *trans*-10 C18:1, *trans*-11 C18:1 and *cis*-9 *trans*-11 C18:2 diet variation was greater than b-cow variation with low repeatability.

**Table 6:** Variance component estimates of milk fatty acids (FA) in dairy cows.

Variable		Estimate	SE	Z value^1^	SD^2^	CV^3^	Rep^4^
Selected FA, g 100g/FA							
C4:0	Diet (Exp)	0.18	0.054	<0.01	0.42	0.13	0.51
	Cow (Exp)	0.04	0.014	<0.01	0.20	0.06	
	Residual	0.04	0.007	<0.01	0.20	0.06	
C6:0	Diet (Exp)	0.11	0.033	<0.01	0.33	0.18	0.48
	Cow (Exp)	0.01	0.005	<0.01	0.12	0.06	
	Residual	0.02	0.003	<0.01	0.12	0.07	
C8:0	Diet (Exp)	0.06	0.018	<0.01	0.24	0.22	0.58
	Cow (Exp)	0.01	0.004	<0.01	0.11	0.10	
	Residual	0.01	0.002	<0.01	0.10	0.09	
C13:0 iso^5^	Diet (Exp)	2.00	0.600	<0.01	0.04	1.51	0.18
	Cow (Exp)	0.30	0.210	0.07	0.02	0.66	
	Residual	1.00	0.200	<0.01	0.04	1.41	
C13:0 anteiso	Diet (Exp)	0.03	0.010	0.01	0.16	14.2	0.15
	Cow (Exp)	0.01	0.005	0.09	0.08	7.23	
	Residual	0.04	0.007	<0.01	0.19	17.1	
C15:0 iso^5^	Diet (Exp)	1.00	0.400	<0.01	0.04	0.17	0.40
	Cow (Exp)	0.30	0.130	0.01	0.02	0.08	
	Residual	0.50	0.100	<0.01	0.02	0.10	
C15:0 anteiso^5^	Diet (Exp)	3.00	0.800	<0.01	0.05	0.12	0.57
	Cow (Exp)	2.00	0.700	<0.01	0.05	0.11	
	Residual	2.00	0.300	<0.01	0.04	0.09	
C15:0^5^	Diet (Exp)	0.02	0.006	<0.01	0.13	0.13	0.27
	Cow (Exp)	0.005	0.0023	0.02	0.07	0.07	
	Residual	0.01	0.002	<0.01	0.11	0.11	
C16:0 iso^5^	Diet (Exp)	0.01	0.004	0.03	0.09	0.38	0.02
	Cow (Exp)	0.001	0.0023	0.40	0.02	0.11	
	Residual	0.02	0.005	<0.01	0.15	0.67	
C16:0	Diet (Exp)	13.9	3.60	<0.01	3.45	0.13	0.58
	Cow (Exp)	2.26	0.835	<0.01	1.57	0.06	
	Residual	2.14	0.429	<0.01	1.46	0.05	
C17:0 iso^5^	Diet (Exp)	0.50	0.210	0.01	0.02	0.13	0.17
	Cow (Exp)	0.20	0.130	0.08	0.01	0.08	
	Residual	0.90	0.190	<0.01	0.03	0.18	
C17:0 anteiso^5^	Diet (Exp)	1.00	0.400	0.01	0.03	0.12	0.27
	Cow (Exp)	0.60	0.300	0.02	0.03	0.09	
	Residual	2.00	0.300	<0.01	0.04	0.15	
C17:0^5^	Diet (Exp)	10.00	2.000	<0.01	0.07	0.15	0.47
	Cow (Exp)	0.60	0.210	<0.01	0.02	0.05	
	Residual	0.70	0.110	<0.01	0.03	0.05	
C18:0 iso^5^	Diet (Exp)	0.10	0.020	<0.01	0.01	0.15	0.43
	Cow (Exp)	0.03	0.013	<0.01	0.01	0.11	
	Residual	0.05	0.009	<0.01	0.01	0.12	
C18:0	Diet (Exp)	4.99	1.514	<0.01	2.23	0.21	0.27
	Cow (Exp)	0.39	0.192	0.02	0.63	0.06	
	Residual	1.06	0.210	<0.01	1.03	0.10	
C18:1, trans-10	Diet (Exp)	3.38	0.999	<0.01	1.84	1.81	0.14
	Cow (Exp)	0.08	0.057	0.08	0.28	0.28	
	Residual	0.48	0.093	<0.01	0.69	0.68	
C18:1, trans-11	Diet (Exp)	1.50	0.449	<0.01	1.22	0.64	0.10
	Cow (Exp)	0.03	0.026	0.15	0.17	0.09	
	Residual	0.23	0.045	<0.01	0.48	0.25	
C18:1, cis-9	Diet (Exp)	11.4	3.56	<0.01	3.37	0.18	0.43
	Cow (Exp)	3.01	1.131	<0.01	1.74	0.09	
	Residual	3.95	0.656	<0.01	1.99	0.11	
C18:1, cis-11	Diet (Exp)	0.03	0.008	<0.01	0.16	0.27	0.30
	Cow (Exp)	0.004	0.0018	0.02	0.06	0.10	
	Residual	0.01	0.002	<0.01	0.09	0.15	
C18:2, cis-9 trans-11	Diet (Exp)	0.20	0.063	<0.01	0.45	0.57	0.17
	Cow (Exp)	0.01	0.006	0.12	0.08	0.11	
	Residual	0.05	0.009	<0.01	0.23	0.30	
C18:3, cis-9 cis-12 cis-15^5^	Diet (Exp)	10.0	4.00	<0.01	0.12	0.24	0.46
	Cow (Exp)	2.0	0.60	<0.01	0.04	0.08	
	Residual	2.0	0.40	<0.01	0.05	0.09	
<16-carbon	Diet (Exp)	15.2	4.63	<0.01	3.90	0.15	0.60
	Cow (Exp)	3.76	1.266	<0.01	1.94	0.07	
	Residual	2.53	0.434	<0.01	1.59	0.06	
16-carbon	Diet (Exp)	11.5	3.49	<0.01	3.39	0.11	0.52
	Cow (Exp)	2.25	0.780	<0.01	1.50	0.05	
	Residual	2.10	0.435	<0.01	1.45	0.05	
>16-carbon	Diet (Exp)	49.3	14.78	<0.01	7.02	0.16	0.38
	Cow (Exp)	3.70	1.550	0.01	1.92	0.04	
	Residual	6.16	1.051	<0.01	2.48	0.06	

^1^Probability of Z-value.

^2^Calculated as the square root of the variance component estimate.

^3^Calculated as SD divided by the respective mean value of the variable.

^4^Rep = σ^2^ Cow/(σ^2^ Cow + σ^2^ Residual).

^5^ These FA are reported in mg/ 100 g FA.

### Empirical models–simple regressions

**Fig 1:**
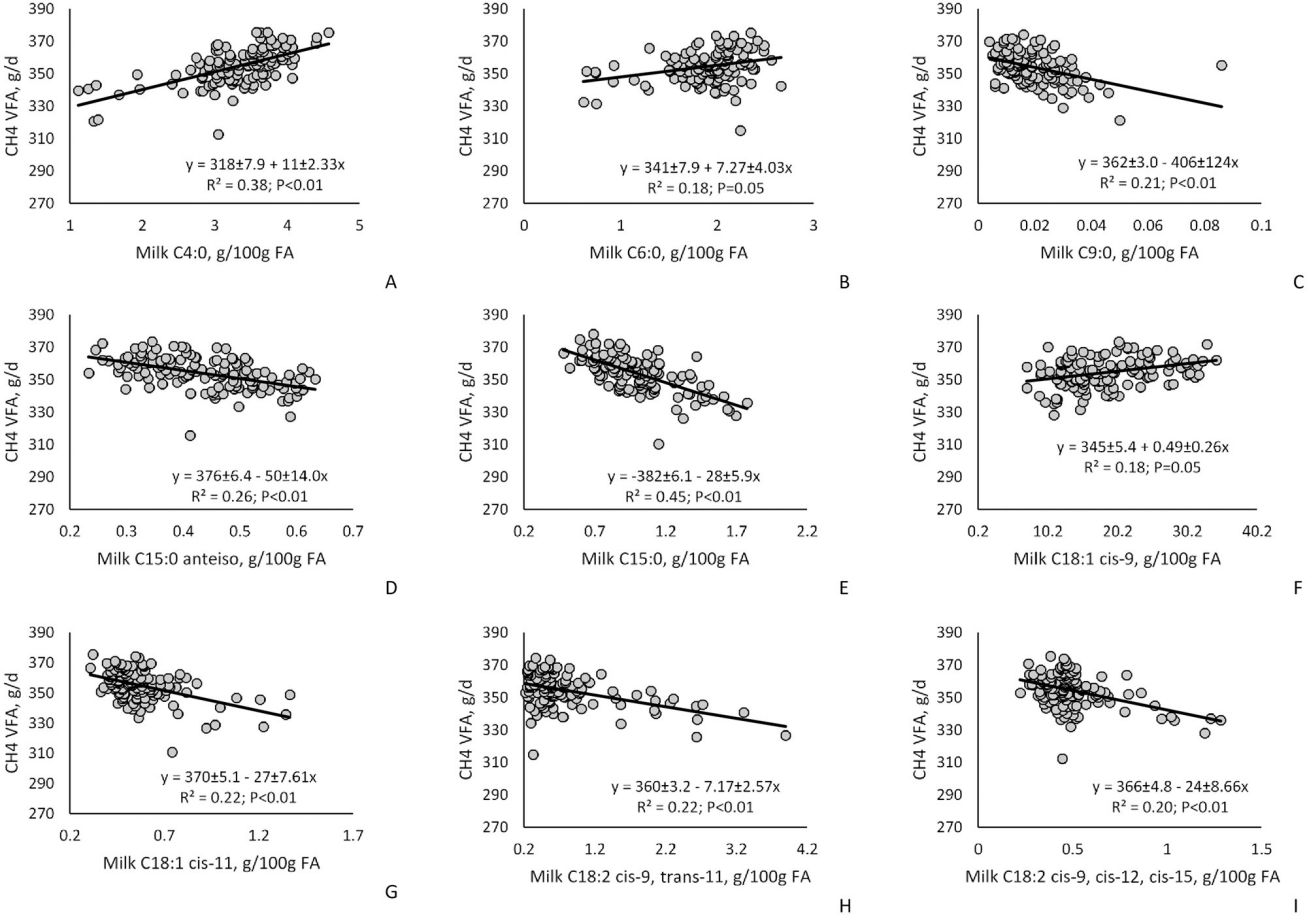
Influence of selected milk fatty acid (FA) on Y_CH_4_VFA estimated by univariate mixed model regression analysis (CH_4_VFA = A + BX_1_) in dairy cows.

Relationships between milk FA and stoichiometric Y_CH_4_VFA are presented in Fig 1 and S2 Table. The C4:0 and C6:0 (*P* < 0.05) were positively related with stoichiometric Y_CH_4_VFA. For the OBCFA, *anteiso* C15:0 (*P* < 0.01) and C15:0 (*P* < 0.01) were negatively associated with stoichiometric CH_4_VFA. Additionally, milk *trans*-11 C18:1 (*P* = 0.02), *cis*-11 C18:1 (*P* < 0.01), *cis*-9, *trans*-11 C18:2 (*P* = 0.01), and *cis*-9, *cis*-12, *cis*-15 C18:3 were negatively related with stoichiometric CH_4_VFA. There was no relationship between the summation of milk FA by source (de novo, mixed and preformed) and stoichiometric CH_4_VFA. We evaluated the relationship between milk OBCFA and rumen VFA (S3 Table). There was no relationship (*P* > 0.05) between both *anteiso* C13:0 and *iso* C13:0 milk FA and concentration of rumen VFA (propionate, valerate, isovalerate, and BCVFA). Milk C15:0 was positively associated with rumen propionate (*P* < 0.01) and valerate (*P* = 0.01). Milk *anteiso* C15:0 was positively associated with rumen propionate (*P* = 0.05), while milk *iso* C15:0 was negatively associated with isovalerate (*P* = 0.01) and BCVFA (*P* = 0.02). Milk *anteiso* C17:0 was positively associated with isovalerate (*P* < 0.01) and BCVFA (*P* = 0.01), while C17:0 was negatively related to with BCVFA (*P* = 0.05).

**Fig 2:**
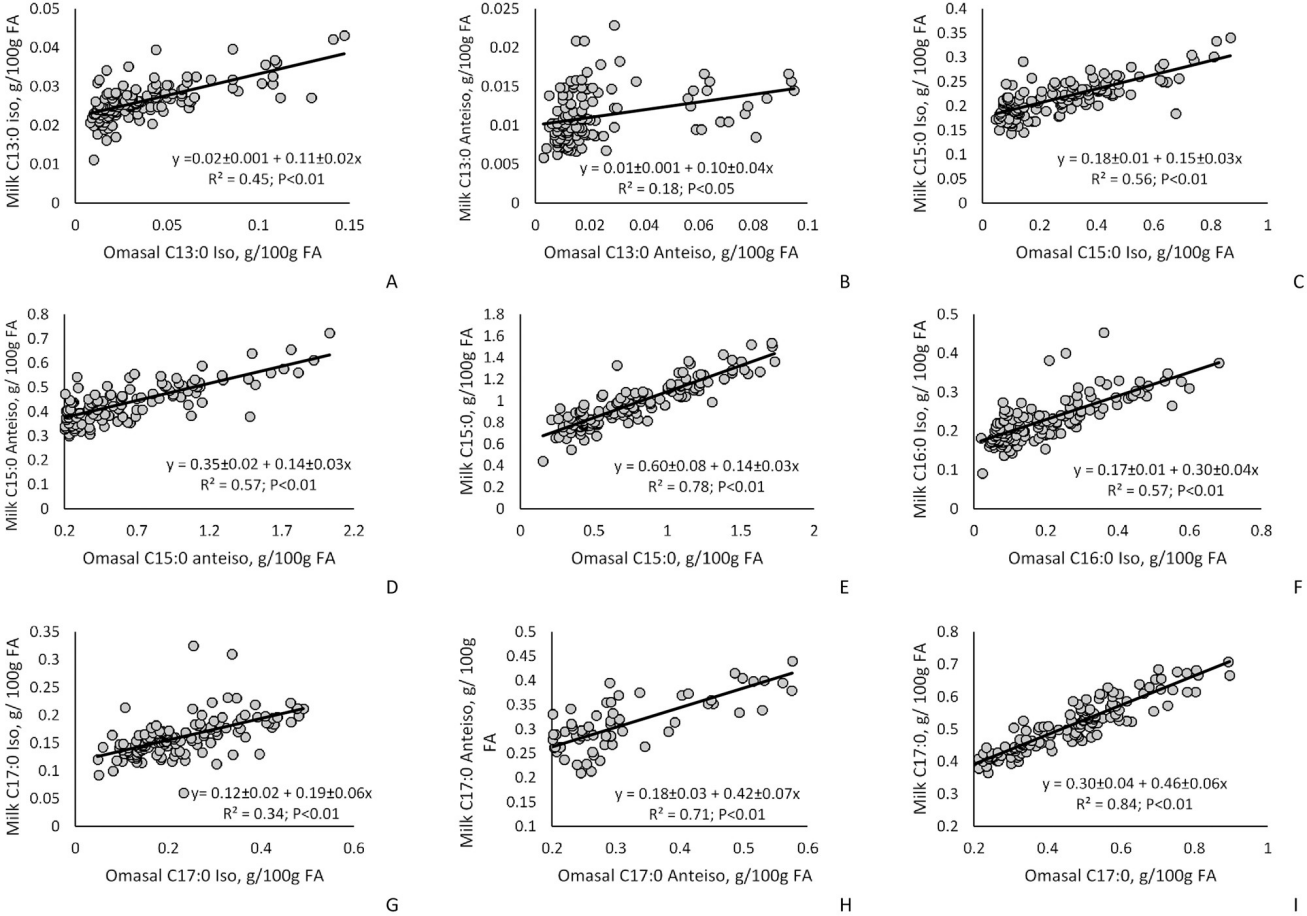
Influence of omasal fatty acid (FA) concentration (g 100 g/ FA) on milk fatty acid concentration (g 100 g/ FA) estimated by univariate mixed model regression analysis (OBCFA = A + BX_1_) in dairy cows.

**Fig 3:**
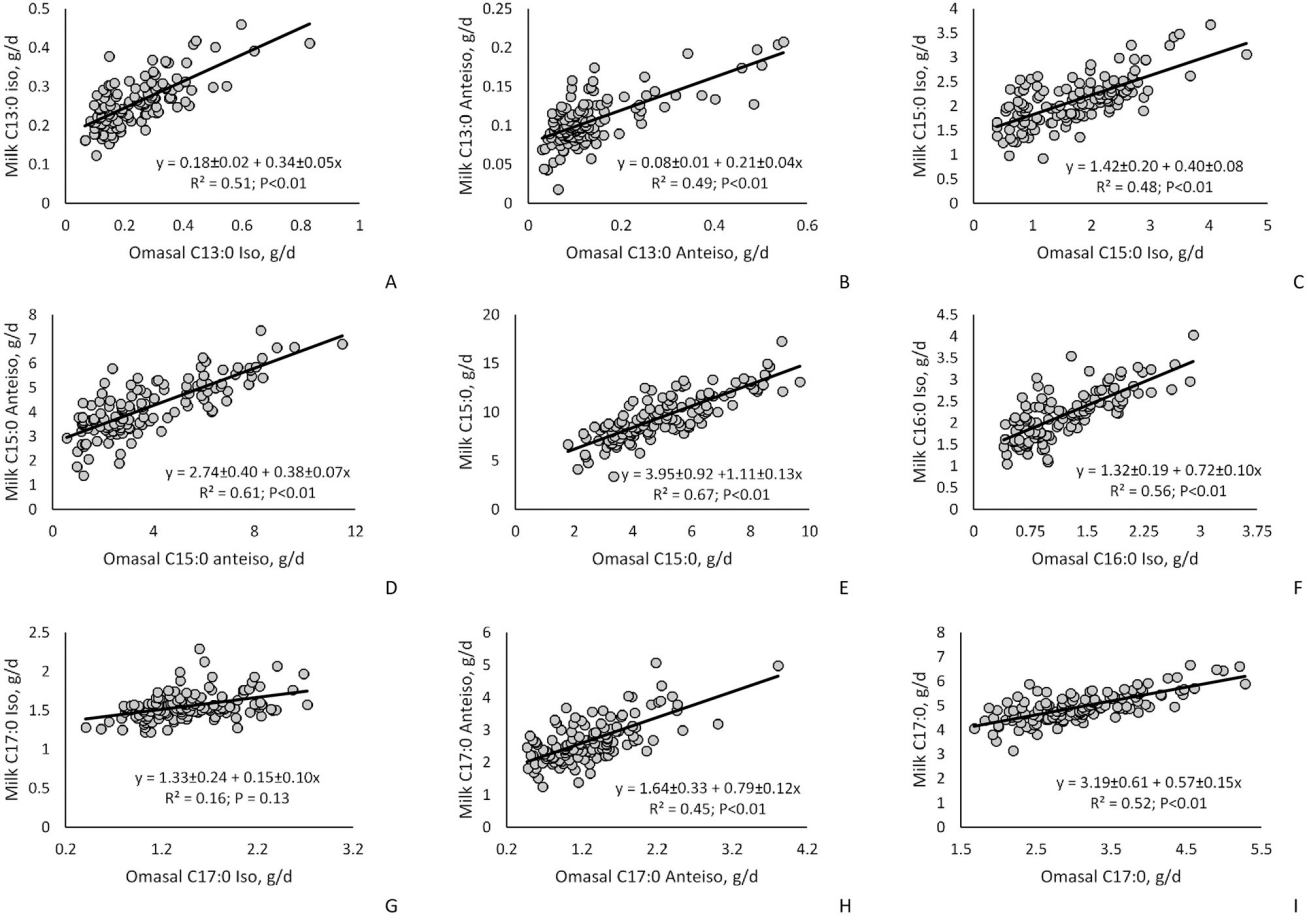
Influence of omasal fatty acid (FA) flow (g/d) on milk fatty acid yield (g/d) estimated by univariate mixed model regression analysis (OBCFA = A + BX_1_) in dairy cows.

The relationship between concentrations and flows of omasal OBCFA on milk FA are presented in Figs 2 and 3. We observed positive relationship between concentration of omasal OBCFA and the concentration of milk OBCFA (*P* < 0.01), as well as positive intercepts for all of the OBCFA evaluated (*P* < 0.01). Similarly, for most OBCFA, we observed positive relationships between omasal flow of OBCFA and yield of milk OBCFA (*P* < 0.01) with exception for milk *iso* C17:0 that was not affected by *iso* C17:0 omasal flow (*P* = 0.13). Regarding the intercept values, for all milk OBCFA, we observed positive intercepts (*P* < 0.01).

### Empirical models–multiple regressions

We evaluated whether calculated ME balance and proportion of omasal FA would affect milk FA profile (Table 7). The concentration of several milk FA was affected by energy balance. Some milk OBCFA (*iso* C13:0, *iso* C15:0, *anteiso* C15:0, and C15:0) were positively associated with the omasal proportion of their respective FA (all *P* < 0.01) and by energy balance (*P* < 0.01). In contrast, the concentration of milk C17:0, *iso* C18:0, C18:0, and *cis*-11 C18:1 were positively influenced by omasal proportion of their respective FA (*P* < 0.01) but negatively associated with energy balance (*P* < 0.05). For milk *cis*-9 C18:1, there was no effect of omasal *cis*-9 C18:1 (*P* = 0.69), but it was inversely related with energy balance (*P* < 0.01). We observed minor effects of DMI associated with rumen VFA on milk OBCFA (S4 Table).

**Table 7:** Influence of energy balance and composition of omasal fatty acids (FA) on milk FA concentration estimated by multivariate mixed model regression analysis (Milk FA = A + BX_1_ + CX_2_) in dairy cows.

Y	A^1^	SE	B^1^	SE	P value	C^1^	SE	P value	Residual
FA, g 100g/FA									
C13:0 iso	0.02	0.001	0.00005	0.000018	0.01	0.12	0.025	<0.01	0.00001
C13:0 anteiso	0.01	0.001	0.00004	0.000027	0.19	0.10	0.036	<0.01	0.0001
C15:0 iso	0.18	0.011	0.0004	0.00013	0.01	0.14	0.030	<0.01	0.001
C15:0 anteiso	0.35	0.025	0.0007	0.00026	0.01	0.14	0.026	<0.01	0.003
C15:0	0.59	0.077	0.002	0.0005	<0.01	0.50	0.067	<0.01	0.010
C16:0 iso	0.17	0.012	0.00004	0.000177	0.81	0.31	0.041	<0.01	0.002
C16:0	15.0	2.77	0.014	0.0108	0.19	1.04	0.214	<0.01	4.29
C17:0 iso	0.12	0.025	0.00013	0.000166	0.45	0.20	0.059	<0.01	0.001
C17:0 anteiso	0.18	0.032	-0.000004	0.0002170	0.98	0.42	0.065	<0.01	0.002
C17:0	0.30	0.033	-0.0005	0.00018	0.01	0.45	0.061	<0.01	0.001
C18:0 iso	0.04	0.004	-0.0002	0.00004	<0.01	0.21	0.057	<0.01	0.0001
C18:0	5.03	1.288	-0.012	0.0063	0.05	0.11	0.022	<0.01	1.44
C18:1, trans-10	-0.01	0.067	0.002	0.0023	0.42	0.54	0.016	<0.01	0.48
C18:1, trans-11	0.30	0.241	-0.002	0.0024	0.48	0.31	0.027	<0.01	0.22
C18:1, cis-9	18.2	2.005	-0.041	0.0132	<0.01	0.14	0.353	0.69	6.31
C18:1, cis-11	0.32	0.056	-0.002	0.0004	<0.01	0.40	0.055	<0.01	0.008
C18:2, cis-9 trans-11	0.76	0.175	0.0008	0.001307	0.56	0.04	0.098	0.72	0.061
C18:3, cis-9 cis-12 cis-15	0.31	0.036	-0.0003	0.00034	0.32	0.18	0.023	<0.01	0.004

^1^A = intercept (All P-values ≤ 0.01).; B = regression coefficient of energy balance; C = regression coefficient of individual omasal FA (same FA as the Y variable).

## Discussion

Lately, several studies have focused on developing reliable and low-cost measures of ruminant enteric CH_4_ emissions on an individual-animal basis. Determining the variability among cows offers the potential for genetic selection of animals that have lower CH_4_ emissions, which is an attractive mitigation strategy because genetic improvements are cumulative and permanent [34]. Milk FA are a promising CH_4_ proxy because of the direct link to microbial digestion in the rumen and energy balance [35]. Additionally, breeding for reduced CH_4_ production has been proposed and therefore indicators of CH_4_ production based on milk FA are of particular interest [9, 36]. A large range in the heritability of CH_4_ production (h^2^: 0.12 to 0.44) estimated using milk FA has been reported [15], even though the R^2^ of the equations were not much different (0.63 to 0.73). In the present study, repeatability and b-cow variation estimated by variance components were used to identify suitable animal variables of rumen fermentation, omasal FA and milk FA related to b-cow differences in estimated CH_4_ emissions. Despite the limited number of observations in our analysis due to our selection criteria focused on the integration of data on fermentation parameters, omasal FA and milk FA, we established important relationships involving animal factors, digestion, omasal flow and milk output related to CH_4_.

A limitation in our study is that we did not have a direct measurement of CH_4_, but rather we used the approach proposed by Wolin [30] to calculate Y_CH_4_VFA. This method could be criticized because it assumes that all fermented substrates have a formula C_6_H_12_O_6_, while some carbohydrates deviate from this general formula. Although this consideration is important, these carbohydrates usually comprise only a small part of ruminant diets [30]. Furthermore, deviations from the C_6_H_12_O_6_ formula could influence variance component of Diet (Exp) but not that of Cow (Exp), which is the major interest of this study. Additionally, the stoichiometric relationships between VFA production and production of H_2_ (substrate for hydrogenotrophic methanogens) suggest that CH_4_ emissions are positively associated with the acetate:propionate ratio in ruminal fluid; however, the relationship between CH_4_ emission and both VFA and pH are variable in the literature and not as straightforward as expected from theory [35]. In our study, the b-cow coefficient of variation (CV) for Y_CH_4_VFA was only 0.01, while the variation in the variance components for diet was 3 times greater than the b-cow variation. Similar to our results, b-cow CV of 0.01, and 0.104 for predicted Y_CH_4_VFA, and total CH_4_ production were previously reported [13]. Between-animal CV in CH_4_ production reported in the literature differ, reflecting differences in feed intake and methodology.

Rumen VFA pattern can be expected to be related to CH_4_ production due to changes in H_2_ balance, such that high acetate and butyrate production enhance CH_4_ production, whereas high propionate production is associated with low CH_4_ emissions [37]. In the present study, b-cow variation in rumen VFA pattern was small (CV ranged from 0.01 to 0.05). Similarly, a previous study reported for sheep a CV for CH_4_ production of 0.098 [38], whereas the CV for molar proportions of acetate, propionate, and butyrate was 0.011, 0.047, and 0.036, respectively. Greater b-cow variation and repeatability for traits such as digestibility, passage, and efficiency of microbial cell synthesis has been previously reported [13] indicating that between-animal variation in CH_4_ may be more closely related to these characteristics than the composition of the rumen microbiome.

The milk fat in ruminants contain greater proportions of saturated FA compared with dietary intake because of extensive biohydrogenation of unsaturated FA in the rumen [39]. During the biohydrogenation of FA, several *trans* FA intermediates are formed under different dietary conditions [40], and therefore they may also be indicators of changes in rumen function. In our study, for both omasal and milk *trans* FA (*trans*-10 C18:1 and *trans*-11 C18:1) diet variation was 5-fold greater than b-cow variation indicating that rumen conditions influencing the synthesis of these FA was more strongly associated with differences in diets than with differences between the cows. Variance components and repeatability of *trans*-10 C18:1 and *trans*-11 C18:1 were similar between omasal flow and milk indicating that these FA are more related with ruminal changes than post-ruminal metabolism. Also, milk *trans*-11 C18:1, *cis*-11 C18:1, *cis*-9, *trans*-11 C18:2, and *cis*-9, *cis*-12, *cis*-15 C18:3 were negatively related with stoichiometric Y_CH_4_VFA. Similarly, a previous study [9] indicated a negative correlation between concentration of *cis*-9, *cis*-12, *cis*-15 C18:3 in milk fat and CH_4_ production. Additionally, milk *trans*-11 C18:1 and *cis*-11 C18:1 were negative correlated with CH_4_ production [10]. Negative association of some unsaturated FA (i.e. *cis*-9 C18:1) with CH_4_ production are expected, especially during negative energy balance where intake and CH_4_ production are low compared with cows in positive energy balance. Also, a negative association of unsaturated FA and CH_4_ is expected since during biohydrogenation some H_2_ are used by rumen bacteria. In addition, increased unsaturated FA in milk may indicate dietary unsaturated fat supplementation and thus decreased intake of fermentable carbohydrates, and or reduce ruminal fermentation of organic matter, and thereby CH_4_ production. Furthermore, several *trans* and *cis* FA occurring in milk fat are biohydrogenation intermediates of both *cis*-9, *cis*-12 C18:2 and *cis*-9, *cis*-12, *cis*-15 C18:3 [39]. Rumen conditions with low fiber and high concentrate diets may induce changes in the extent of biohydrogenation and formation of biohydrogenation intermediates [40]. With reduced rumen pH, the predominant biohydrogenation pathway of *cis*-9, *cis*-12 C18:2 may shift to the *trans*-10 pathway [40]. Therefore, these observations explain the negative correlation obtained between the concentration of some milk *trans* FA and CH_4_ production, whereas diet factors are more strongly related to the differences in these FA than to between-animal differences.

OBCFA are suggested to reflect rumen function including ruminal fermentation pattern, duodenal flow of microbial protein and acidosis [6]. Overall for OBCFA omasal flow the variation associated with diet was considerably greater than the between-cow variation with low repeatability. Furthermore, in our study, we observed weak associations between rumen VFA profile and milk *iso* and *anteiso* OBCFA. Similarly, a previous study observed that rumen and milk OBCFA responses were minimal following infusion of large amounts of VFA (acetate, propionate and isovalerate) and suggested that shifts in ruminal OBCFA are primarily affected by altered populations of different rumen microbial strains driven by dietary composition as opposed to altered VFA available in the extracellular space for FA synthesis [41]. In the rumen, de novo FA in bacteria are synthesized by two types of FA synthetases: straight-chain and branched-chain FA synthetase [42]. Linear odd-chain FA are formed when propionyl-CoA, instead of acetyl-CoA, is used as a primer [42]. In our study, we observed that milk C15:0 was positively associated with rumen propionate and valerate, which agrees with previous findings suggesting that C15:0 and C17:0 are formed through elongation of propionate or valerate [6]. Additionally, when we considered DMI in the equations, we also observed a positive effect of propionate and DMI on milk C15:0, while for C17:0 a positive relationship with valerate and DMI was detected. Similar to our results, [43] reported milk concentrations of C15:0 and the sum of C17:0 and *cis*-9 C17:1 to be positively related to propionate concentration in the rumen. Since it is expected that propionate production is negatively related to CH_4_ production, this suggests a negative relationship between the concentration of these OBCFA in milk and CH_4_ production. In the present study, we did not observe an association between the proportion of omasal C15:0 and C17:0 and Y_CH_4_VFA, while milk C15:0 was negatively related to CH_4_VFA. Similarly, the results from previous studies have been equivocal and reporting negative correlations between milk C15:0 and C17:0 and CH_4_ production [44] or no significant relationships between these FA with CH4 yield [10].

Importantly, when we evaluated the effect of omasal OBCFA (g/100 g of FA) on their respective milk OBCFA (g/100 g of FA) and the omasal flow of OBCFA (g/d) on their respective yield of milk OBCFA (g/d), we detected positive intercepts, which may indicate endogenous synthesis or elongation in the mammary gland. Similar to our results, a previous study reported greater secretion of C15:0, C17:0, and *iso* C17:0 in milk fat than could be accounted for by intestinal absorption [45]. In the mammary gland, endogenous chain elongation using propionyl-CoA as precursor [46] explains the occurrence of certain odd-chain FA (i.e. C5:0, C7:0, C9:0 and C11:0) in milk and it may also increase the amount of other odd-chain FA transferred from the duodenum (C13:0, C15:0 and C17:0) into milk. Also, milk secretion of *iso* C17:0 and *anteiso* C17:0 in excess of duodenal flows of those FA has been also observed indicating synthesis in tissues [47]. Limited synthesis of the *iso* 17:0 has also been reported [7], and methodological issues due to coelution of *cis*-9 C16:1 with anteiso C17:0 [48] are also possible factors that affect these differences between omasal flow and milk FA secretion. In addition to mammary gland, other tissues have also been shown to have the ability to synthesize OBCFA from propionate [49] and, therefore, OBCFA that are present in milk in greater amounts than their respective duodenal flow could partially be a result of the synthesis in the mammary gland and increasing amounts of OBCFA mobilized from other tissues. Repeatability was lower for *iso* C13:0, *iso* C15:0, and *iso* C17:0 omasal flow compared to milk output. Additionally, we observed that milk *iso* C13:0 and *iso* C15:0 were positively associated to rumen propionate concentration and feed intake, which in turn suggest that potentially other factors can affect the output of these OBCFA in milk. Therefore, the endogenous synthesis of OBCFA, elongation of some OBCFA into their longer chain equivalents, and synthesis in other tissues may limit their use as biomarkers of rumen function and CH_4_ proxy.

Additionally, we observed that energy balance is an important factor influencing milk FA profile. For milk OBCFA, *iso* C13:0, *iso* C15:0, *anteiso* C15:0, and C15:0 were positively influenced by omasal proportion of their respective FA and by energy balance. Similar to our results, Craninx et al. [50] reported that OBCFA with chain lengths of 14 or 15 carbon atoms showed an increasing pattern as lactation period progressed and cows entered in positive energy balance, whereas OBCFA with chain lengths of 17 carbon atoms showed the opposite pattern of response. In dairy cows, *cis*- 9 C18:1, C18:0 and C16:0 are the main FA present in adipose tissue [51]. During early lactation, mobilization of body reserves of fat increases the circulation of these FA and their uptake by the mammary gland. Therefore, the decrease in the concentration of these OBCFA in milk fat may be a dilution effect since other long-chain FA will increase during periods of negative energy balance. Therefore, some of the inconsistency when predicting CH_4_ using concentration of milk FA can be explained by energy balance and lactation stage, both being factors that can influence the relationship between milk FA and CH_4_ emission.

The short- and medium-chain FA (4 to 14 carbons) and a portion of the 16-carbon FA are derived from de novo synthesis from acetate and to a lesser extent BHBA [40]. Therefore, since acetate and butyrate production in the rumen is associated with H_2_ production, some de novo milk FA may be a proxy for CH_4_. In contrast to our expectation, we found weak relationships between most de novo milk FA and Y_CH_4_VFA, with only the concentration of milk C4:0 and a tendency for C6:0 being positively associated with Y_CH_4_VFA. A positive correlation between de novo FA and CH_4_ (g/d) has been reported [9], while others reported that C12:0, and C14:0 were positively associated with CH_4_ (g/d) [52]. Also, for de novo milk FA C4:0, C6:0 and C8:0 and for C16:0 the diet variation was 3-fold higher than the b-cow variation, but repeatability for these FA was high. Also, a previous study reported that the concentration of C16:0 in milk fat was moderately positively related to CH_4_ yield (g/kg of DMI), and concentrations of C6:0, C8:0, and C10:0 in milk fat tended to be weakly positively related to CH_4_ yield [10]. Although diet is still the major factor impacting the variance components of de novo FA, these FA seem more promising as proxies for CH_4_ parameters as heritability for short and medium chain FA are greater than those for mixed and unsaturated milk FA [53]. However, selecting animals for low C4:0 to C12:0 milk FA may result in lower CH_4_, but also may reduce milk fat content and yield due to the correlation between milk de novo FA concentration and these traits.

Although we estimated CH_4_ using VFA stoichiometry rather than directly measuring CH_4_ our b-cow estimates are in line with previous reports. Additionally, we generated b-cow estimates for *trans*-FA and OBCFA that have been related with rumen function, but our analysis of omasal flow of FA and animal factors suggest that factors, such as feed intake and energy balance, should be considered because they are likely associated to post-ruminal changes in the appearance of these FA into milk fat. Additionally, a recent study used the equations from the meta-analysis of van Lingen et al. [10] to quantify the CH_4_ emissions traits predicted by selected milk FA and to assess their main sources of variation [54]. They reported wide variability in estimated CH_4_ emissions traits among different farms within dairy system (TMR fed vs. hay + concentrate) indicating that factors related with feeding management and other animal management practices are likely related to CH_4_ emissions [54]. Feeding management factors (i.e. feeding frequency, bunk space, etc.) influence feed intake, slug-feeding, rumen pH and animal behavior [55], which in turn may affect CH_4_ emissions. Although we did not characterize and have available data in our data set regarding feed management, we cannot rule out the possibility that factors that influence feed behavior may impact CH_4_ estimates.

## Conclusion

Our findings demonstrate that for most omasal and milk FA examined, a larger variation can be attributed to dietary factors than b-cow differences with low to moderate repeatability. Even though we observed that some milk FA were positively or negatively associated with Y_CH_4_VFA, other factors such as energy balance had a pronounced effect on these estimates. Therefore, this may preclude the utilization of milk FA as a proxy for CH_4_ predictions. Based on our dataset, between-animal variation in milk FA profile was small, which may suggest that caution should be exercised when using milk FA to select low-emitting animals in breeding programs. Because of the greater between-diet variability compared with between-animal variation for most milk FA, they may be used as a proxy for detecting differences between diets and farms; however, these differences can also be predicted by empirical models.

## Supporting information

S1 Table Data sources and characteristics of included studies. (DOCX)

S2 Table Influence of milk fatty acid (FA) on stoichiometry methane (CH_4_VFA) estimated by univariate mixed model regression analysis (CH_4_VFA = A + BX_1_) in dairy cows. (DOCX)

S3 Table Influence of rumen VFA on the concentration of milk odd- and branched-chain fatty acids (OBCFA) (g 100 g/ FA), estimated by univariate mixed model regression analysis (OBCFA = A + BX_1_) in dairy cows. (DOCX)

S4 Table Influence of rumen VFA, and DMI on milk odd- and branched-chain fatty acids (OBCFA), estimated by bivariate mixed model regression analysis (OBCFA = A + BX_1_ + BX_2_) in dairy cows. (DOCX)

S1 Data (XLSX)
