# Supplementary material for: Between-cow variation in milk fatty acids associated with methane production
Source: PLoS One. 2020 Aug 6;15(8):e0235357. doi: 10.1371/journal.pone.0235357 (PMC7410208; doi:10.1371/journal.pone.0235357)
Supplement: S1 Table — (DOCX) [file pone.0235357.s001.docx]

Supplementary Table 1. Data sources and characteristics of included studies.

| Study | Reference | N | Number of treatments | Diet/Treatment description |
| --- | --- | --- | --- | --- |
| 1 | [16, 17] | 10 | 2 | Treatments comprised Strip grazing grass or barn fed hay grass. |
|  |  |  |  |  |
|  |  |  |  |  |
|  |  |  |  |  |
| 2 | [16, 17] | 15 | 3 | Treatments evaluated the effects of feeding diets based on hay, grass silage with no additive, or formic acid-based additive |
|  |  |  |  |  |
|  |  |  |  |  |
|  |  |  |  |  |
| 3 | [18, 19] | 16 | 4 | Treatments comprised total mixed rations offered ad libitum containing 600 g of forage/kg of diet dry matter, with red clover silage replacing grass silage in a ratio of 0:100, 33:67, 67:33, and 100:0 on a dry matter basis. |
|  |  |  |  |  |
|  |  |  |  |  |
|  |  |  |  |  |
| 4 | [20, 21] | 16 | 4 | Treatments comprised a basal diet containing no additional oil (control), or supplemented with 200 g of fish oil, 200 g of fish oil and 500 g of sunflower oil, or 200 g of fish oil and 500 g of linseed oil. |
|  |  |  |  |  |
|  |  |  |  |  |
|  |  |  |  |  |
| 5 | [22, 23] | 16 | 4 | Treatments comprised fish oil supplementation at 0, 75, 150, and 300 g/d. |
|  |  |  |  |  |
|  |  |  |  |  |
|  |  |  |  |  |
| 6 | [24] | 16 | 4 | Treatments comprised total mixed rations based on grass silage with a forage:concentrate ratio of 35:65 or 65:35 containing 0 or 50 g/kg sunflower oil. |
|  |  |  |  |  |
|  |  |  |  |  |
|  |  |  |  |  |
| 7 | [25] | 16 | 4 | Treatments comprised of total mixed rations based on grass silage with a forage:concentrate ratio of 60:40 supplemented with 0 or 50 g/kg of rapeseed oil as pure rapeseed oil, whole rapeseed or crushed rapeseed. |
|  |  |  |  |  |
|  |  |  |  |  |
|  |  |  |  |  |
| 8 | Unpublished | 16 | 4 | Treatments comprised total mixed rations based on grass silage with a forage:concentrate ratio of 80:20, 60:40, 40:60 or 20:80 supplemented with 30 g/kg of linseed oil. |
|  |  |  |  |  |
|  |  |  |  |  |
|  |  |  |  |  |
| 9 | Unpublished | 16 | 4 | Treatments comprised of total mixed rations based on grass silage with forage to concentrate ratio of 65:35 or 35:65 supplemented with fiber or starch rich concentrates. |
|  |  |  |  |  |
|  |  |  |  |  |
|  |  |  |  |  |
