# Supplementary material for: Between-cow variation in milk fatty acids associated with methane production
Source: PLoS One. 2020 Aug 6;15(8):e0235357. doi: 10.1371/journal.pone.0235357 (PMC7410208; doi:10.1371/journal.pone.0235357)
Supplement: S2 Table — (DOCX) [file pone.0235357.s002.docx]

Supplementary Table 2. Influence of milk fatty acid (FA) on stoichiometry methane (CH_4_VFA) estimated by univariate mixed model regression analysis (CH_4_VFA = A + BX_1_) in dairy cows

| X_1_ | A^1,2^ | SE | B^1^ | SE | P value | Residual |
| --- | --- | --- | --- | --- | --- | --- |
| Milk FA, g/ 100 g FA |  |  |  |  |  |  |
| C4:0 | 318 | 7.9 | 11 | 2.33 | <0.01 | 94 |
| C6:0 | 341 | 7.9 | 7.25 | 4.034 | 0.05 | 108 |
| C8:0 | 355 | 6.2 | -0.51 | 5.311 | 0.92 | 108 |
| C9:0 | 362 | 3.0 | -406 | 124.5 | <0.01 | 108 |
| C13:0 iso | 349 | 6.4 | 197 | 228.1 | 0.39 | 108 |
| C13:0 anteiso | 355 | 2.7 | -46 | 130.3 | 0.72 | 108 |
| C15:0 iso | 360 | 6.9 | -26 | 29.8 | 0.39 | 105 |
| C15:0 anteiso | 376 | 6.4 | -50 | 14.0 | <0.01 | 93 |
| C15:0 | 382 | 6.1 | -28 | 5.9 | <0.01 | 89 |
| C16:0 iso | 353 | 5.2 | 4.88 | 20.022 | 0.81 | 108 |
| C16:0 | 345 | 8.8 | 0.33 | 0.310 | 0.29 | 106 |
| C17:0 iso | 359 | 3.5 | -28 | 17.0 | 0.10 | 106 |
| C17:0 anteiso | 362 | 4.8 | -30 | 15.8 | 0.06 | 107 |
| C17:0 | 361 | 8.7 | -14 | 16.5 | 0.41 | 107 |
| C18:0 iso | 345 | 5.5 | 171 | 92.9 | 0.07 | 103 |
| C18:0 | 345 | 5.6 | 0.87 | 0.473 | 0.07 | 107 |
| C18:1, trans-10 | 355 | 2.4 | -1.28 | 1.000 | 0.20 | 110 |
| C18:1, trans-11 | 359 | 3.2 | -2.62 | 1.073 | 0.02 | 106 |
| C18:1, cis-9 | 345 | 5.4 | 0.49 | 0.263 | 0.05 | 107 |
| C18:1, cis-11 | 370 | 5.1 | -27 | 7.61 | <0.01 | 105 |
| C18:2, cis-9 trans-11 | 360 | 3.2 | -7.17 | 2.579 | 0.01 | 104 |
| C18:3, cis-9 cis-12 cis-15 | 366 | 4.8 | -24 | 8.76 | 0.01 | 108 |
| de novo^3^ | 363 | 8.5 | -0.33 | 0.318 | 0.30 | 105 |
| Mixed^3^ | 342 | 9.8 | 0.41 | 0.317 | 0.20 | 104 |
| Preformed^3^ | 355 | 8.5 | -0.02 | 0.185 | 0.89 | 107 |

^1^A = intercept; B = regression coefficient; X_1_ = variable.

^2^ All P-values ≤ 0.01.

^3^ De novo FA originate from mammary de novo synthesis (<16 carbons), preformed FA originated from extraction from plasma (>16 carbons), and mixed FA originate from both sources.
