# Supplementary material for: Between-cow variation in milk fatty acids associated with methane production
Source: PLoS One. 2020 Aug 6;15(8):e0235357. doi: 10.1371/journal.pone.0235357 (PMC7410208; doi:10.1371/journal.pone.0235357)
Supplement: S3 Table — (DOCX) [file pone.0235357.s003.docx]

Supplementary Table 3. Influence of rumen VFA on the concentration of milk odd- and branched-chain fatty acids (OBCFA) (g 100 g/ FA), estimated by univariate mixed model regression analysis (OBCFA = A + BX_1_) in dairy cows

| Y | X_1_^1^ | A^2^ | SE | B^2^ | SE | P value | Residual |
| --- | --- | --- | --- | --- | --- | --- | --- |
| Milk FA, g/ 100 g FA |  |  |  |  |  |  |  |
| C13:0 anteiso | BCVFA | 0.01 | 0.004 | 0.00006 | 0.00018 | 0.73 | 0.00006 |
| C13:0 anteiso | iVal | 0.01 | 0.003 | 0.00001 | 0.00022 | 0.98 | 0.00006 |
| C13:0 anteiso | Prop | 0.01 | 0.003 | -0.000001 | 0.00001 | 0.97 | 0.00001 |
| C13:0 anteiso | Val | 0.01 | 0.004 | 0.00022 | 0.00025 | 0.39 | 0.00006 |
| C13:0 iso | BCVFA | 0.03 | 0.003 | -0.00015 | 0.00012 | 0.21 | 0.00002 |
| C13:0 iso | iVal | 0.03 | 0.002 | -0.00023 | 0.00015 | 0.13 | 0.00002 |
| C13:0 iso | Prop | 0.03 | 0.004 | 0.000004 | 0.00002 | 0.86 | 0.00002 |
| C13:0 iso | Val | 0.02 | 0.003 | 0.00023 | 0.00019 | 0.23 | 0.00002 |
| C15:0 | BCVFA | 1.11 | 0.108 | -0.0059 | 0.0039 | 0.13 | 0.01301 |
| C15:0 | iVal | 1.09 | 0.095 | -0.0078 | 0.0049 | 0.11 | 0.01302 |
| C15:0 | Prop | 0.58 | 0.131 | 0.0022 | 0.00059 | <0.01 | 0.01123 |
| C15:0 | Val | 0.79 | 0.105 | 0.015 | 0.0058 | 0.01 | 0.01237 |
| C15:0 anteiso | BCVFA | 0.43 | 0.045 | 0.00006 | 0.0019 | 0.98 | 0.00330 |
| C15:0 anteiso | iVal | 0.41 | 0.037 | 0.00154 | 0.0023 | 0.51 | 0.00323 |
| C15:0 anteiso | Prop | 0.32 | 0.060 | 0.0006 | 0.00030 | 0.05 | 0.00308 |
| C15:0 anteiso | Val | 0.38 | 0.044 | 0.0039 | 0.0028 | 0.17 | 0.00324 |
| C15:0 iso | BCVFA | 0.26 | 0.020 | -0.0022 | 0.00091 | 0.02 | 0.00078 |
| C15:0 iso | iVal | 0.25 | 0.016 | -0.0029 | 0.0011 | 0.01 | 0.00079 |
| C15:0 iso | Prop | 0.19 | 0.030 | 0.00014 | 0.00015 | 0.38 | 0.00078 |
| C15:0 iso | Val | 0.22 | 0.021 | 0.00009 | 0.0014 | 0.95 | 0.00080 |
| C17:0 | BCVFA | 0.57 | 0.037 | -0.0026 | 0.0013 | 0.05 | 0.00127 |
| C17:0 | iVal | 0.55 | 0.033 | -0.0030 | 0.0016 | 0.06 | 0.00128 |
| C17:0 | Prop | 0.47 | 0.047 | 0.00025 | 0.00020 | 0.23 | 0.00126 |
| C17:0 | Val | 0.54 | 0.038 | -0.0019 | 0.0020 | 0.36 | 0.00129 |
| C17:0 anteiso | BCVFA | 0.18 | 0.040 | 0.0046 | 0.0016 | 0.01 | 0.00228 |
| C17:0 anteiso | iVal | 0.17 | 0.034 | 0.0082 | 0.0019 | <0.01 | 0.00208 |
| C17:0 anteiso | Prop | 0.34 | 0.054 | -0.00035 | 0.00026 | 0.18 | 0.00259 |
| C17:0 anteiso | Val | 0.26 | 0.041 | 0.0013 | 0.0024 | 0.59 | 0.00253 |
| C17:0 iso | BCVFA | 0.16 | 0.030 | -0.00004 | 0.0012 | 0.97 | 0.00116 |
| C17:0 iso | iVal | 0.16 | 0.025 | 0.00024 | 0.0014 | 0.87 | 0.00116 |
| C17:0 iso | Prop | 0.19 | 0.039 | -0.00013 | 0.00018 | 0.47 | 0.00117 |
| C17:0 iso | Val | 0.15 | 0.032 | 0.0011 | 0.0020 | 0.58 | 0.00115 |

^1^ Prop= Propionate (mmol/mol); Val = Valerate (mmol/mol); iVal = Isovalerate (mmol/mol); BCVFA = Isovalerate + Isobutyrate.

^2^A = intercept (All P-values ≤ 0.01.); B = regression coefficient of X_1_ variable.
