# Supplementary material for: Between-cow variation in milk fatty acids associated with methane production
Source: PLoS One. 2020 Aug 6;15(8):e0235357. doi: 10.1371/journal.pone.0235357 (PMC7410208; doi:10.1371/journal.pone.0235357)
Supplement: S4 Table — (DOCX) [file pone.0235357.s004.docx]

Supplementary Table 4. Influence of rumen VFA, and DMI on milk odd- and branched-chain fatty acids (OBCFA), estimated by bivariate mixed model regression analysis (OBCFA = A + BX_1_ + BX_2_) in dairy cows

| Y | A^1^ | SE | P value | B^2^ | SE | P value | X_2_^2,3^ | C^2^ | SE | P value |
| --- | --- | --- | --- | --- | --- | --- | --- | --- | --- | --- |
| Milk FA, g/ 100 g FA |  |  |  |  |  |  |  |  |  |  |
| C13:0 anteiso | 0.01 | 0.010 | 0.22 | 0.0002 | 0.00027 | 0.37 | BCVFA | 0.0002 | 0.00025 | 0.33 |
| C13:0 anteiso | 0.01 | 0.010 | 0.23 | 0.0002 | 0.00027 | 0.38 | iBut | 0.0007 | 0.00061 | 0.23 |
| C13:0 anteiso | 0.02 | 0.009 | 0.11 | 0.0002 | 0.00027 | 0.43 | iVal | 0.0002 | 0.00031 | 0.55 |
| C13:0 anteiso | 0.01 | 0.003 | 0.01 | 0.0001 | 0.00009 | 0.22 | Prop | -0.0001 | 0.00001 | 0.92 |
| C13:0 anteiso | 0.01 | 0.009 | 0.34 | 0.0002 | 0.00026 | 0.54 | Val | 0.0008 | 0.00037 | 0.03 |
| C13:0 iso | 0.04 | 0.013 | 0.03 | 0.0002 | 0.00032 | 0.62 | BCVFA | 0.0001 | 0.00029 | 0.79 |
| C13:0 iso | 0.03 | 0.013 | 0.06 | 0.0002 | 0.00031 | 0.49 | iBut | 0.0010 | 0.00070 | 0.15 |
| C13:0 iso | 0.04 | 0.013 | 0.01 | 0.0001 | 0.00032 | 0.73 | iVal | -0.0002 | 0.00037 | 0.67 |
| C13:0 iso | 0.02 | 0.005 | 0.004 | 0.0004 | 0.00015 | 0.01 | Prop | 0.0000002 | 0.00002 | 0.99 |
| C13:0 iso | 0.05 | 0.013 | 0.007 | 0.0001 | 0.00031 | 0.63 | Val | -0.0005 | 0.00044 | 0.22 |
| C15:0 | 0.63 | 0.158 | 0.004 | 0.0091 | 0.00348 | 0.01 | BCVFA | 0.0006 | 0.00325 | 0.86 |
| C15:0 | 0.50 | 0.154 | 0.01 | 0.0101 | 0.00338 | 0.004 | iBut | 0.0154 | 0.00771 | 0.05 |
| C15:0 | 0.69 | 0.149 | 0.002 | 0.0084 | 0.00347 | 0.02 | iVal | -0.0032 | 0.00408 | 0.43 |
| C15:0 | 0.33 | 0.140 | 0.05 | 0.0148 | 0.00380 | 0.0002 | Prop | 0.0020 | 0.00055 | 0.001 |
| C15:0 | 0.58 | 0.150 | 0.005 | 0.0090 | 0.00337 | 0.01 | Val | 0.0047 | 0.00497 | 0.35 |
| C15:0 anteiso | 0.39 | 0.195 | 0.08 | 0.0093 | 0.00438 | 0.04 | BCVFA | 0.0034 | 0.00408 | 0.40 |
| C15:0 anteiso | 0.43 | 0.190 | 0.05 | 0.0087 | 0.00434 | 0.05 | iBut | 0.0039 | 0.00992 | 0.70 |
| C15:0 anteiso | 0.40 | 0.184 | 0.06 | 0.0092 | 0.00436 | 0.04 | iVal | 0.0043 | 0.00509 | 0.40 |
| C15:0 anteiso | 0.21 | 0.067 | 0.01 | 0.0069 | 0.00198 | 0.001 | Prop | 0.0005 | 0.00028 | 0.083 |
| C15:0 anteiso | 0.46 | 0.185 | 0.04 | 0.0085 | 0.00429 | 0.05 | Val | 0.0005 | 0.00623 | 0.93 |
| C15:0 iso | 0.24 | 0.088 | 0.03 | 0.0026 | 0.00213 | 0.23 | BCVFA | 0.0004 | 0.00197 | 0.84 |
| C15:0 iso | 0.23 | 0.085 | 0.03 | 0.0026 | 0.00210 | 0.22 | iBut | 0.0020 | 0.00479 | 0.68 |
| C15:0 iso | 0.25 | 0.082 | 0.02 | 0.0025 | 0.00212 | 0.24 | iVal | 0.0001 | 0.00246 | 0.97 |
| C15:0 iso | 0.15 | 0.034 | 0.002 | 0.0025 | 0.00103 | 0.02 | Prop | 0.0001 | 0.00015 | 0.48 |
| C15:0 iso | 0.32 | 0.083 | 0.005 | 0.0025 | 0.00205 | 0.22 | Val | -0.0051 | 0.00294 | 0.09 |
| C17:0 | 0.46 | 0.059 | <.0001 | 0.0008 | 0.00131 | 0.54 | BCVFA | -0.0001 | 0.00123 | 0.97 |
| C17:0 | 0.42 | 0.057 | <.0001 | 0.0011 | 0.00128 | 0.39 | iBut | 0.0039 | 0.00293 | 0.18 |
| C17:0 | 0.47 | 0.055 | <.0001 | 0.0006 | 0.00131 | 0.64 | iVal | -0.0012 | 0.00154 | 0.45 |
| C17:0 | 0.51 | 0.051 | <.0001 | -0.0029 | 0.00135 | 0.03 | Prop | 0.0003 | 0.00020 | 0.15 |
| C17:0 | 0.40 | 0.056 | <.0001 | 0.0008 | 0.00125 | 0.50 | Val | 0.0038 | 0.00186 | 0.05 |
| C17:0 anteiso | 0.15 | 0.067 | 0.060 | -0.0001 | 0.00182 | 0.94 | BCVFA | 0.0037 | 0.00169 | 0.03 |
| C17:0 anteiso | 0.19 | 0.065 | 0.02 | -0.0007 | 0.00184 | 0.72 | iBut | 0.0056 | 0.00427 | 0.20 |
| C17:0 anteiso | 0.17 | 0.062 | 0.02 | -0.0003 | 0.00181 | 0.87 | iVal | 0.0044 | 0.00211 | 0.04 |
| C17:0 anteiso | 0.34 | 0.063 | 0.001 | 0.0001 | 0.00185 | 0.96 | Prop | -0.0004 | 0.00026 | 0.18 |
| C17:0 anteiso | 0.27 | 0.062 | 0.002 | -0.0010 | 0.00183 | 0.60 | Val | -0.0025 | 0.00262 | 0.34 |
| C17:0 iso | 0.21 | 0.051 | 0.004 | 0.0006 | 0.00134 | 0.65 | BCVFA | 0.0009 | 0.00128 | 0.48 |
| C17:0 iso | 0.20 | 0.050 | 0.004 | 0.0006 | 0.00133 | 0.66 | iBut | 0.0031 | 0.00326 | 0.35 |
| C17:0 iso | 0.22 | 0.047 | 0.002 | 0.0005 | 0.00134 | 0.70 | iVal | 0.0007 | 0.00159 | 0.68 |
| C17:0 iso | 0.18 | 0.045 | 0.0033 | 0.0002 | 0.00129 | 0.89 | Prop | -0.0001 | 0.00018 | 0.46 |
| C17:0 iso | 0.26 | 0.047 | 0.001 | 0.0005 | 0.00131 | 0.71 | Val | -0.0027 | 0.00206 | 0.19 |

^1^A = intercept; All P-values ≤ 0.01.

^2^B = regression coefficient of DMI; C = regression coefficient of X_2_ variable.

^3^ Prop= Propionate (mmol/mol); Val = Valerate (mmol/mol); iVal = Isovalerate (mmol/mol); BCVFA = Isovalerate + Isobutyrate.
